# Association of In-person vs Virtual Education With Community COVID-19 Case Incidence Following School Reopenings in the First Year of the COVID-19 Pandemic

**DOI:** 10.1001/jamanetworkopen.2023.8300

**Published:** 2023-04-14

**Authors:** Meredith Matone, Xi Wang, Deanna Marshall, Jing Huang, Diana Worsley, Corinne Filograna, David Rubin

**Affiliations:** 1 PolicyLab, Children’s Hospital of Philadelphia, Philadelphia, Pennsylvania; 2 Department of Pediatrics, Perelman School of Medicine at the University of Pennsylvania, Philadelphia; 3 Leonard Davis Institute of Health Economics at University of Pennsylvania, Philadelphia; 4 Department of Biostatistics, Epidemiology, and Informatics, Perelman School of Medicine at the University of Pennsylvania, Philadelphia; 5 Clinical Futures, Children’s Hospital of Philadelphia, Philadelphia, Pennsylvania; 6 Formerly PolicyLab, Children’s Hospital of Philadelphia, Philadelphia, Pennsylvania; 7 Merck and Company, Rahway, New Jersey

## Abstract

**Importance:**

The variability in timing of middle and secondary school reopenings during the 2020 to 2021 school year in the US presents an opportunity to examine the associations of different approaches to in-person education with changes in community COVID-19 incidence. Early studies on this topic have reached mixed conclusions and may be biased by unmeasured confounders.

**Objective:**

To estimate the association of in-person vs virtual instruction for students at the sixth grade level or above with county-level COVID-19 incidence in the first year of the COVID-19 pandemic.

**Design, Setting, and Participants:**

This cohort study included matched pairs of counties resuming school programs with in-person vs virtual instruction, drawn from 229 US counties that contained a single public school district and with county populations exceeding 100 000 residents. Counties that contained 1 single public school district and reopened in-person schooling for students at the sixth grade level or above during the fall of 2020 were matched 1-to-1 with counties whose school district reopened with only virtual instruction, based on geographic proximity, population-level demographic factors, the resumption of school district–level fall sports activity, and baseline county COVID-19 incidence rates. Data were analyzed from November 2021 to November 2022.

**Exposures:**

In-person instruction for students at the sixth grade level or above resuming between August 1 and October 31, 2020.

**Main Outcomes and Measures:**

County-level daily COVID-19 incidence per 100 000 residents.

**Results:**

The inclusion criteria and subsequent matching algorithm led to the identification of 51 pairs of matched counties among 79 total unique counties. Exposed counties had a median (IQR) of 141 840 (81 441-241 910) residents each, and unexposed counties had a median (IQR) of 131 412 (89 011-278 666) residents each. County schools with in-person vs virtual instruction had similar daily COVID-19 case incidence within the first 4 weeks after in-person reopening, but counties with in-person instruction had higher daily incidence beyond 4 weeks. Daily case incidence per 100 000 residents among counties with in-person instruction, compared with counties with virtual instruction, was higher at 6 weeks (adjusted incidence rate ratio, 1.24 [95% CI, 1.00-1.55]) and at 8 weeks after (adjusted incidence rate ratio, 1.31 [95% CI, 1.06-1.62]). This outcome was also concentrated in counties where schools provided full rather than hybrid instructional models.

**Conclusions and Relevance:**

In a cohort study of matched pairs of counties that reopened with in-person vs virtual instruction at the secondary school level in the 2020 to 2021 academic year, counties with in-person school instructional models early in the COVID-19 pandemic experienced increases in county-level COVID-19 incidence at 6 and 8 weeks after in-person reopening, compared with counties with virtual instructional models.

## Key Points

**Question:**

Was resuming in-person vs virtual instruction for middle and high school students in the fall of 2020 associated with differences in COVID-19 community incidence?

**Findings:**

In a cohort study of 51 matched pairs of counties that reopened with in-person vs virtual instruction, those that reopened with in-person schooling experienced slightly higher county-level COVID-19 incidence at 6 and 8 weeks after in-person reopening compared with those that reopened virtually.

**Meaning:**

These findings of this cohort study should be interpreted in the context of competing concerns of reduced school engagement and social well-being of children in prolonged virtual learning environments.

## Teaser

This cohort study estimates the association of in-person vs virtual instruction for students at the sixth grade or above with county-level COVID-19 incidence in the first year of the COVID-19 pandemic.

## Introduction

Arguably one of the most debated aspects of the COVID-19 pandemic in the US has been the safety of schools to children, teachers, and families and, by extension, the contribution of in-person instruction to community transmission. While early evidence after March 2020 demonstrated that children and adolescents were at lowest risk of serious symptomatic infection from SARS-CoV-2, children and adolescents have represented an increasing proportion of cases over time. Decisions on the resumption of in-person instruction for the fall 2020 semester were largely informed by new knowledge that older youth could transmit the virus and by considerations of school staff safety and resources available for mitigation in the period prior to the availability of a vaccine. Decisions were varied with some public school districts delaying fall resumption of in-person instruction, particularly in the secondary school (high school) setting, while other school districts reopened for in-person instruction, either fully for all students or with hybrid models that reduced daily in-person participation.

The variability in timing for school reopenings in the 2020 to 2021 school year presents an opportunity to examine the association of these different approaches to in-person vs virtual instruction of students in secondary school classrooms with changes in community COVID-19 incidence during the early fall of 2020. Early research has suggested that in-person instruction at the primary and secondary levels is associated with increases in community transmission at the county-level in the US but that this association is modest in size and influenced by school mitigation policies.^1,2^ However, empirically studying the contribution of in-person instruction to community transmission has been challenging for several reasons. Most significant has been unobserved confounding related to highly dynamic and variable school-community engagement with mitigation strategies that can be very difficult to measure over time.^3^ Poor measurement of mitigation strategies and adherence with such mitigation can confound ecological and observational studies designed to describe the association between schools and community transmission. The few studies that have characterized the association of school closures or in-person reopenings with community transmission have not controlled for simultaneous nonpharmaceutical interventions at the community level that may have confounded their observed findings.^3,4,5^ In this study, we use a matched-pair design to estimate the longitudinal association of in-person schooling models with county-level COVID-19 transmission as measured in fall of 2020. Using data from a collated resource of school mitigation practices, we identified pairs of counties that reopened for in-person education on time (whether fully or with hybrid in-person models) vs those that delayed in-person instructional models at the onset of the 2020 to 2021 school year. Counties were matched within close proximity geographically on a set of school and community-level covariates and mitigation practices to reduce the influence of confounding, thereby providing stronger evidence on the association between early approaches to in-person education at the secondary level and subsequent COVID-19 community transmission.

## Methods

This cohort study used county-level publicly available data and was therefore determined to be exempt from formal review and informed consent by the institutional review board of Children’s Hospital of Philadelphia. This study followed the Strengthening the Reporting of Observational Studies in Epidemiology (STROBE) reporting guideline for cohort studies.

### Counties Selection

Our sample included US counties that contain a single public school district and met at least 1 of the following criteria: (1) contained a state capital; (2) had at least 1 city with population exceeding 100 000 persons; (3) represented the most populous county within the state; (4) had a county population density greater than 250 persons per square mile; or (5) registered mean daily new case counts within the county of 20 or more between June 23 and July 6, 2020. These criteria mirror methods used in other county-level studies of COVID-19 transmission across the pandemic and reflect the need to identify counties with sufficient population size to reduce uncertainty in community incidence rate estimates.^6,7^ Based on these inclusion criteria, 229 counties were included in the initial study sample before matching.

### Exposure

Counties included in the exposed group must have opened for in-person schooling for at least 1 grade of students at the sixth grade level or above between August 1 and October 31, 2020. We chose to focus on sixth grade or above given that higher reported rates of SARS-CoV-2 infection among older children led to greater variability in reopening plans than among elementary school–age children, in whom reported SARS-CoV-2 infection rates were, at the time, much lower. Data on school reopening plans were obtained from a systematic scan of official school district website contents, including announcements, COVID-19 reopening plan documents, and school district press releases. In some instances, official school district–run social media accounts were used as sources for up-to-date announcements on school reopening dates and policies. For counties with staggered reopening for different grades, the reopening date was considered the first date that at least 1 grade level sixth or above was eligible to return. The method for data abstraction in this study included a double coding by 2 team members and a secondary validation of a random sample of 20 of 79 school districts (25%) in the sampling frame.

### Matching Criteria and Process

Unexposed counties were eligible for matching if they did not reopen in-person schooling for students in sixth grade or above before December 31, 2020, or reopened for in-person instruction at least 4 weeks after the matched exposed county’s reopening date. Among exposed counties and eligible unexposed counties, we first performed exact matching on geographic proximity. Eligible geographic matches were defined as within the same US Bureau of Economic Analysis region or US Census metropolitan statistical area designations, or within 200 miles of straight-line distance between counties. We then manually performed 1:1 matching with replacement to find an unexposed county that was closest to the exposed county on the following characteristics: total population, population density, the presence of school district–level fall sports activity (which has been considered an independent risk factor associated with community transmission),^8,9^ weekly median reproduction number (Rt, a measure of instantaneous reproduction number that has been used to model time-varying changes in transmission in SARS-CoV-2 and other viruses),^10^ and weekly case incidence per 100 000 county residents for the week prior to exposed county’s school reopening. Details of the data sources and matching thresholds for each matching variable are provided in eTable 1 in Supplement 1.

### Outcome

The primary outcome was the daily incidence of new COVID-19 cases per 100 000 residents in each county. When delayed or batch or backlogged reporting of daily case counts were observed,^11^ the daily incidence data for COVID-19 were smoothed using 3-day rolling mean of reported case counts. The preintervention baseline period was 2 weeks prior to school reopening date (time zero), and the postintervention observation period was an 8-week period after the school reopening date.

### Covariates

County-level characteristics that are potentially associated with both school reopening (the exposure) and community COVID-19 incidence (the outcome) were included as confounding factors based on existing literature.^6,12^ The time-invariant covariates included the proportion of county residents at less than 200% of the poverty level, the proportion of residents with diabetes, and a measure of mask wearing in the general population.^13^

The time-varying covariates included the temperature and social distancing or mobility measures (in sensitivity analyses only) in each county on each day during the observation time-period. Details on data source, definition, and operationalization of each covariate are provided in eTable 2 in Supplement 2.

### Statistical Analysis

The analytic data set contained daily longitudinal data of each county. Before analysis, we first excluded outliers, defined as days with estimated Rt outside of the 1st to 99th percentile (0.27 and 2.71) in the observation period. We fit a generalized linear mixed-effect model (GLMM) to evaluate the association between school reopening and daily COVID-19 incidence per 100 000 county residents. The GLMM used a log-link function to account for the skewed distribution of daily case incidence and adjusted for time-invariant and time-varying covariates. Piecewise linear terms were used to fit the nonlinear association of wet-bulb temperatures with daily case incidence, following the methods of Jing et al.^6^ Hierarchical random intercept and slope were used to account for correlations within matched pairs of counties and the correlations within longitudinal measures of individual county, separately.

The time variable in the GLMM was calculated as the difference between the date of COVID-19 case identification and the date of school reopening. To allow for nonlinear trajectories of community transmission over time, the GLMM included cubic effects of time using the B-spline method. Interaction terms between school reopening and time were included to detect differential trends in community transmission.

Because the GLMM used a log link function of daily case incidence, the effect estimates were expressed as ratios of daily case incidence between exposed and unexposed counties, after adjusting for covariates. To examine case incidence differences over the interval, we also estimated model-projected daily case incidences under 2 counterfactual scenarios: a setting in which all counties reopened school (ie, the exposure variable was set to 1 for all counties in the GLMM model) vs a setting in which no county reopened school (ie, the exposure variable was to 0 for all counties). The median and IQR of model-projected daily case incidences across all counties under these 2 scenarios are presented in graphs.

#### Stratified Analyses

We conducted 2 sets of stratified analyses to assess association modification by (1) full vs partial in-person education schedules and (2) baseline COVID-19 community incidence rates.^3,14^ Full opening was defined as 5 days per week of in-person instruction with no virtual component, and partial opening was defined as openings that staggered in-person education for smaller cohorts within the district (eg, hybrid learning models that included every other in-person day schedules, alternating weekly in-person schedules, and/or staggered grade-level re-entry into in-person learning). The stratified analysis by baseline COVID-19 community incidence rates was intended to weigh relative value of Centers for Disease Control and Prevention recommendations of community incidence thresholds for conducting school operations for in-person learning in the 2020 to 2021 school year.^15^ The low or moderate baseline risk stratum included pairs in which the exposed counties had a mean of fewer than 20 daily cases per 100 000 county residents in the week before school reopening, and exposed counties in the high baseline risk strata had a mean of 20 or more daily cases per 100 000 persons in the week before school reopening.

#### Sensitivity Analyses

As an additional robustness check, we conducted multiple sensitivity analyses that considered different subgroups of counties and adjusted for concomitant social distancing mitigation requirements in communities over time. Further details on sensitivity analyses are provided in the eMethods in Supplement 1.

Analyses were conducted using R statistical software version 4.0.3 (R Project for Statistical Computing). *P* values were 2-sided, and statistical significance was set at *P* = .05. Data were analyzed from November 2021 to November 2022.

## Results

### Balanced Baseline Characteristics Between Matched Pairs

The inclusion criteria and subsequent matching algorithm led to the identification of 51 pairs of matched counties among 79 total unique counties. Exposed counties had a median (IQR) of 141 840 (81 441-241 910) residents each, and unexposed counties had a median (IQR) of 131 412 (89 011-278 666) residents each. Table 1 presents the balanced distributions between the matched counties with or without school reopening on all matching variables, including geographic, demographic- and school district–level characteristics, and disease transmission levels at baseline. Most counties (45 exposed counties [88.2%] and 47 unexposed counties [92.2%]) were in the Southeastern US. The median (IQR) weekly COVID-19 incidence during the week before school reopening date was 122.3 (69.1-167.2) cases per 100 000 county residents in the exposed counties and 110.7 (74.0-180.1) cases per 100 000 county residents in the matched unexposed counties. Matched county pairs had comparable baseline social distancing and seasonal temperatures.

**Table 1.** Comparison of County Demographics, Disease Transmission, and Social Distancing Measures at Baseline Between Matched Counties With or Without School Reopening^a^

Characteristics of counties	Median (IQR)	
	Counties with schools reopened (n = 51)	Counties without schools reopened (n = 51)
Demographic and geographic characteristics		
Total population	141 840 (81 441 to 241 910)	131 412 (89 011 to 278 666)
Population density, persons per mi^2^	358.3 (164.9 to 694.5)	379.2 (188.1 to 735.8)
Population age <18 y, %	22.8 (21.0 to 25.1)	24.3 (21.8 to 24.9)
Children in public schools, %	84.9 (82.5 to 87.1)	81.2 (77.1 to 87.1)
Population enrolled in undergraduate college, %	18.6 (15.9 to 23.9)	21.4 (17.5 to 24.6)
Region, No. (%)		
Far West	1 (2.0)	1 (2.0)
Great Lakes	1 (2.0)	0
Mideast	4 (7.8)	3 (5.9)
Southeast	45 (88.2)	47 (92.2)
Baseline community transmission		
Median Rt during 1 wk before school reopening date	0.88 (0.75 to 1.03)	0.83 (0.74 to 0.96)
Weekly new COVID-19 cases per 100 000 residents in the week before school reopening date	122.3 (69.1 to 167.2)	110.6 (74.0 to 180.1)
Districts with active Fall sports, No. (%)	36 (70.6)	36 (70.6)
Social distancing in the week before school reopening date, mean, %^b^	−11.1 (−19.7 to −3.2)	−11.7 (−20.3 to −5.1)
Wet-bulb temperature, in the week before school reopening date, mean, °F	72.5 (69.1 to 74.5)	71.5 (70.5 to 75.7)

Abbreviation: Rt, COVID-19 instantaneous reproduction number.

^a^ Baseline was defined as 2 weeks before school reopening.

^b^ Social distancing level was measured by the percentage change in visitation to nonessential venues compared with a pre–COVID-19 period. Essential venues (eg, food stores, pharmacies) and nonessential venues (eg, cinemas and theaters, spas and hair salons, hotels, restaurants) were categories based on guidelines issued by various state governments and policy makers, and visitation to nonessential venues was measured using daily cellphone movement provided by Unacast. The change in visitation to nonessential venues was calculated by comparing the daily visitation during observation period with the mean visitations in a 4-week prepandemic baseline period (February 10 to and March 8, 2020) in each county.

### Differences in Daily COVID-19 Incidence Between Matched Pairs During the 8 Weeks After School Reopening

Table 2 presents the adjusted incidence rate ratios (aIRRs) of daily COVID-19 incidence between exposed and unexposed counties on different days after school reopening. Exposed counties had similar daily COVID-19 incidence as the matched unexposed counties in the first 4 weeks after in-person reopening. On day 14 after reopening, the difference was not statistically significant (aIRR, 1.06 [95% CI, 0.89-1.28]). However, the difference in daily case incidence between exposed and unexposed counties increased beyond 4 weeks. The daily case incidence per 100 000 residents in the exposed counties continued to increase compared with the unexposed counties at 42 days after reopening (aIRR, 1.24 [95% CI, 1.00-1.55]) and at 56 days after reopening (aIRR, 1.31 [95% CI, 1.06-1.62]).

**Table 2.** Daily COVID-19 Incidence per 100 000 Individuals Between Matched Counties With or Without School Reopening, in All Matched Pairs and in Strata By Mode of Reopening and by Baseline Risk

Counties included in the analysis	Adjusted incidence rate ratio (95% CI), time from reopening, d^a^				*P* value^b^
	14	28	42	56	
Among all matched pairs of counties (51 pairs, 79 counties)	1.06 (0.89-1.28)	1.15 (0.93-1.42)	1.24 (1.001-1.55)	1.31 (1.06-1.62)	.02
Stratified analysis by model of school reopening^c^					
Full opening (22 pairs; 36 counties)	1.03 (0.77-1.37)	1.15 (0.81-1.62)	1.32 (0.91-1.90)	1.52 (1.05-2.19)	.02
Hybrid (29 pairs; 49 counties)	1.16 (0.92-1.46)	1.22 (0.93-1.60)	1.25 (0.94-1.65)	1.23 (0.94-1.63)	.13
Stratified analysis by baseline community transmission level^d^					
Low or moderate baseline risk (30 pairs; 53 counties)	1.09 (0.87-1.36)	1.14 (0.88-1.48)	1.21 (0.92-1.58)	1.29 (0.994-1.68)	.28
High baseline risk (21 pairs; 34 counties)	1.05 (0.78-1.40)	1.14 (0.81-1.60)	1.22 (0.86-1.73)	1.24 (0.89-1.73)	.21

^a^ The estimates were from generalized linear mixed-effect models. The models adjusted for population density, percentage of population younger than age 18 years, rate of poverty, use of masks in public (percentage of people reporting to always wear mask in public when expecting to be within 6 feet of another person, in a survey during July 2-14, 2020), and daily wet-bulb-temperature (3-14 days lagged rolling mean). The generalized linear mixed-effect models used a log link function to account for the skewed distribution of daily cases in each county. Hierarchical random intercept and slope were used to account for correlations within matched pairs of counties and the correlations within longitudinal measures of individual county separately.

^b^ *P* values obtained from testing for null hypothesis of no significant difference in trajectory of COVID-19 incidence over time between exposed and unexposed counties, using Wald test on the regression coefficients of 3 interactions among school reopening and polynomials of time (linear, quadratic, and cubic time).

^c^ The data on model of school reopening was obtained through a systematic scan of official school district websites, including superintendent announcements, official district reopening plan documents, and official school district press releases. In some instances, official school district websites linked to district-run social media accounts for up-to-date announcements on school reopening dates and policies.

^d^ Low or moderate baseline risk was defined as exposed counties had fewer than 20 mean daily cases per 100 000 persons in the week before school reopening; high baseline risk, exposed counties had 20 or more mean daily cases per 100 000 persons in the week before school reopening.

The Figure shows the model-estimated daily case incidence per 100 000 residents under 2 counterfactual scenarios assuming all counties having or not having in-person instruction. The model-estimated daily case incidence in the setting of all counties having virtual instruction remained lower than those in the setting of all counties having resumed in-person instruction, particularly later in the observation period: on day 28 after reopening, the median (IQR) estimated daily incidence was 13.3 (10.8-18.3) cases per 100 000 residents if all counties had used virtual instruction and 15.4 (12.4-20.8) cases per 100 000 residents if all counties had used in-person instruction (Figure). On day 56 after reopening, the median (IQR) estimated daily incidence was 13.2 (9.4-19.3) cases per 100 000 residents if all counties had been virtual and 17.4 (12.4-25.3) cases per 100 000 residents if all counties had used in-person instruction.

**Figure.**
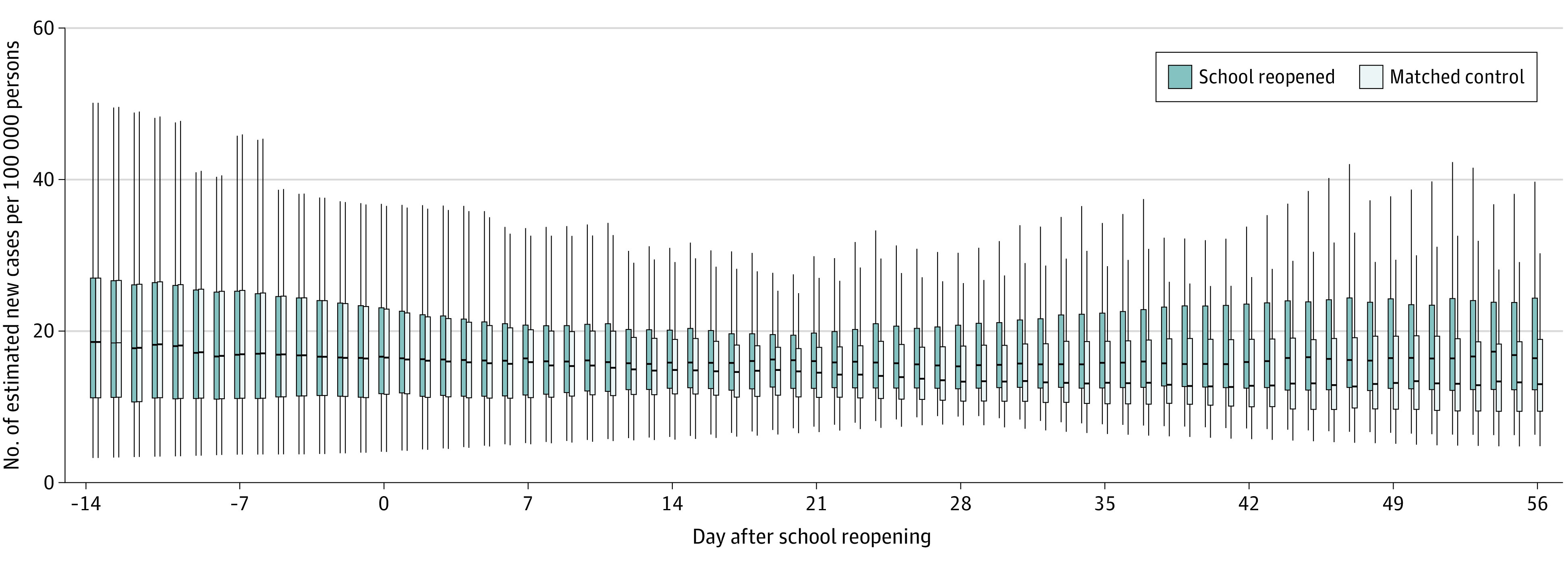
Adjusted Estimated Daily COVID-19 Incidence per 100 000 Individuals, Assuming All Counties Either Reopened or Did Not Reopen In-person Schools The boxplot shows the distributions of model-estimated daily COVID-19 case counts per 100 000 persons in the 51 matched pairs of counties under counterfactual scenarios, with blue boxplots representing all counties reopening their schools, and gray boxplots representing all counties not reopening their schools. The box represents the IQR of the estimates with the middle line representing the median of the distribution. The whiskers extend to 1.5 times the IQR. Time is aligned at the school reopening day (time 0). The estimates were derived from a generalized linear mixed effect model, which adjusted for population density, percentage of population younger than 18 years, rate of poverty, use of masks in public (percentage of people reporting to always wear mask in public when expecting to be within 6 feet of another person), and daily wet-bulb temperature (3-14 days lagged rolling means).

In stratified analyses (Table 2), differences were of larger magnitude among matched pairs with counties that reopened fully for in-school education. By 56 days after in-person reopening and compared with schools that remained fully virtual, daily incidence per 100 000 residents was higher in the counties with a full reopening (aIRR, 1.52 [95% CI, 1.05-2.19]) and in counties using a hybrid in-person/virtual model (aIRR, 1.23 [95% CI, 0.94-1.63]).

When stratifying matched pairs by the baseline community transmission level of exposed counties, we did not observe significant differences between strata (low or moderate baseline risk vs high baseline risk). In sensitivity analyses, the main estimates were robust to further adjustments of social distancing measures and in subgroups of the sample (eTable 3 and eTable 4 in Supplement 1).

## Discussion

In this matched cohort study of school reopenings at the secondary school level in the 2020 to 2021 academic year, we found in-person education (both hybrid and full instructional models) were associated with lagged increases in county-level case incidence of COVID-19 at 6 and 8 weeks after in-person reopening. The association of full in-person reopenings with subsequent community incidence at 8 weeks was also stronger than the association observed for hybrid reopenings and was robust to the inclusion of other community measures of social distancing and mitigation during the period. The magnitude of estimated increase in daily case incidence attributable to in-person vs virtual instruction was small and remained near 2 daily cases per 100 000 individuals at 4 weeks and 4 daily cases per 100 000 individuals at 8 weeks after school reopening.

This study sought to build a more rigorous approach to examining the association between school reopenings and COVID-19 community transmission early in the pandemic by addressing limitations related to comparison groups, confounding of other cooccurring mitigation policies, or insufficient geographic samples. With a highly sophisticated matched design with multiple sensitivity analyses inclusive of enriched data on school and community factors, this study affirms findings of prior studies that detected a positive association between in-person school reopenings and community transmission.^1,2,5^ That this increase was fairly small may suggest that that adherence to mitigation strategies within schools that resumed in-person education might have been sufficient to narrow the difference in community case incidence between schools that reopened in-person vs virtually. A developing body of evidence, for example, has suggested that masking guidelines for students, faculty, and staff, in particular, may have reduced transmission in school communities following reopening.^2,16,17^

The magnitude of school contribution to community transmission found in this study must be interpreted in the context of the potential benefits of in-person instruction models on the academic, social, mental health, and physical outcomes of many students. In addition to education, schools offer essential health services and social supports, such as chronic disease management, school meals, and identification and protection in suspected cases of child abuse. While nascent, the evidence on virtual school has suggested associations of virtual learning with negative physical, mental, and emotional health for some students.^18,19^ The suspension of in-person instruction has also been associated with learning loss and decline in reading and mathematics scores during the pandemic.^20,21^ While the emerging evidence suggests that many children experienced worsened outcomes during the pandemic, it is difficult to disentangle the relative contribution of virtual instruction among the many cooccurring factors impacting children, their families, and their communities. Additionally, for some students, virtual instruction may have mitigated preexisting challenges with in-school learning.^22,23,24^

### Limitations

The study has some limitations. Unobserved practices in school mitigation that were not ascertainable in existing data sources as well as unknown enforcement of mitigation strategies across districts during this period may introduce unobserved confounding and threaten the inference of the observed association. The greater association of full vs hybrid instructional plans during school reopening with subsequent community transmission, for example, could plausibly be explained by more crowded classrooms and insufficient physical distancing during periods of substantial transmission. But those outcomes could also be a proxy for less enforcement in schools that opened hastily or a proxy for wider enforcement challenges outside of school in communities that may have been more lax with public health recommendations. Similarly, although a strength of this study is its inclusion of factors measuring mobility, social distancing, and school mitigation policies regarding fall sports programs, the available data sources were still insufficient to measure informal gatherings within homes. It is plausible that in-home gatherings may have varied by school instructional model (eg, informal multihousehold child care arrangements among families in districts with virtual instruction). Nonrandom variability in the frequency of in-home gatherings may partially explain study findings, although it remains uncertain in which direction this association might have been. Data collection for this study is challenging to reproduce, given the time-variant nature of school website documentation and the heterogeneity in school communication channels during the pandemic period (eg, formal policy documentation housed on school district website vs social media posting of school information on a district-based media account). Future studies of time-variant school policies may consider documentation strategies that include screen captures of websites at the time of ascertainment.

There are other limitations related to generalizability. The results are only generalizable to secondary school reopenings and reflect a period in which population immunity from natural infection was low, vaccinations were not yet available, and public attention to mitigation in community and school settings was higher than later periods in the pandemic. The consistency of these findings following the circulation of more transmissible variants and the introduction of vaccinations warrants further investigation. Additional studies will also need to consider the contribution of within-school mitigation practices, such as masking, which at that time were universal, but which became optional in many schools over time. Moreover, the study sample is heavily weighted by the experience within the US South during this period. The reason is 2-fold: (1) summer and fall 2020 in-person school reopenings disproportionately occurred in southern states and (2) the analysis focused on single-district counties to ensure clean ascertainment of the school exposure context at the county-level. While the selection of single district counties represents a methodologic strength in reducing mismeasurement of exposures (opening dates, school safety protocols, sports initiation), it contributed to geographic skew in the study sample, as single-district counties are also more likely to be found in southern states.

## Conclusions

In this cohort study of school reopenings at the secondary school level in the 2020 to 2021 academic year, we found a modest association between in-person secondary school instruction and community COVID-19 transmission early in the pandemic. The implications for future public health preparedness include consideration of the relatively small and manageable magnitude of school contribution to community transmission that may present a tolerable risk for the resumption of in-person education, with sufficient mitigation measures. This is particularly important in the context of the potential influence of virtual instructional models on learning loss, socialization, and emotional well-being among children. As such, these data can provide context in a broader debate about the relative value of different approaches to public health mitigation in schools should another similar pandemic respiratory virus emerge in the future.

## Notes

Supplement 1. **eTable 1.** Matching Variables **eTable 2.** Covariates Adjusted in Regression Models **eMethods.** Sensitivity Analyses **eTable 3.** Sensitivity Analyses: Ratios of Daily COVID-19 Incidence per 100 000 Individuals Between Matched Counties With or Without School Reopening, in Subgroups of Matched Pairs **eTable 4.** Ratios of Daily COVID-19 Incidence per 100 000 Individuals Between Matched Counties With or Without School Reopening, in Models Further Adjusting for Social Distancing and Mobility Measures

Supplement 2.
Data Sharing Statement
