# Supplementary material for: Association of In-person vs Virtual Education With Community COVID-19 Case Incidence Following School Reopenings in the First Year of the COVID-19 Pandemic
Source: JAMA Netw Open. 2023 Apr 14;6(4):e238300. doi: 10.1001/jamanetworkopen.2023.8300 (PMC10105309; doi:10.1001/jamanetworkopen.2023.8300)
Supplement: Supplement 1. — eTable 1. Matching Variables eTable 2. Covariates Adjusted in Regression Models eMethods. Sensitivity Analyses eTable 3. Sensitivity Analyses: Ratios of Daily COVID-19 Incidence per 100 000 Individuals Between Matched Counties With or Without School Reopening, in Subgroups of Matched Pairs eTable 4. Ratios of Daily COVID-19 Incidence per 100 000 Individuals Between Matched Counties With or Without School Reopening, in Models Further Adjusting for Social Distancing and Mobility Measures [file jamanetwopen-e238300-s001.pdf]

Supplemental Online Content

Matone M, Wang X, Marshall D, et al. Association of in-person vs virtual education with community COVID-19 case incidence following school reopenings in the first year of the COVID-19 pandemic. *JAMA Netw Open*. 2023;6(4):e238300. doi:10.1001/jamanetworkopen.2023.8300

eTable 1. Matching Variables
eTable 2. Covariates Adjusted in Regression Models
eMethods. Sensitivity Analyses
eTable 3. Sensitivity Analyses: Ratios of Daily COVID-19 Incidence per 100 000 Individuals Between Matched Counties With or Without School Reopening, in Subgroups of Matched Pairs
eTable 4. Ratios of Daily COVID-19 Incidence per 100 000 Individuals Between Matched Counties With or Without School Reopening, in Models Further Adjusting for Social Distancing and Mobility Measures

This supplemental material has been provided by the authors to give readers additional information about their work.

© 2023 Matone M et al. *JAMA Network Open*.

eTable 1. Matching Variables

Matching variable	Data source	Threshold for comparability
Date of reopening for in-person schooling for students in 6th grade or above	Data on school reopening plans were obtained from a systematic scan of official school district website contents, including announcements, COVID-19 reopening plan documents, and school district press releases. In some instances, official school district-run Twitter or Facebook accounts were used as sources for up-to-date announcements on school reopening dates and policies.	Unexposed counties were eligible for matching if they did not reopen for in-person schooling for students in 6th grade or above before January 2021 or reopened at least 4 weeks after the matched exposed county's reopening date.
**Geographic area**	US. Bureau of Economic Analysis (BEA) US Census metropolitan statistical area (MSA) designations	The matched unexposed county must be within the same BEA region or MSA as the exposed county, or within 200 miles of straight-line distance of the exposed county.
**Total population**	American Community Survey	After matching on other variables, an eligible unexposed county that was closest to the exposed county on total population was chosen.
**Population density**	American Community Survey	After matching on other variables, an eligible unexposed county that was closest to the exposed county on population density was chosen.
**Weekly case incidence per 100,000 county residents for the week prior to exposed county's school reopening**	USAFacts	The difference in weekly cases per 100,000 residents at the match date between the exposed county and the matched unexposed county must be below 108, a threshold chosen based on the interquartile range of weekly cases across the sample for August 1 to December 31, 2020.
**Weekly median reproduction number (Rt) for the week prior to exposed county's school reopening**	The daily Rt in each county was estimated using the method of Cori et al.[a] with a moving average window of 3 days and an average of generation time of SARS-CoV-2 assumed as 7.5 (SD 3.4) days	The difference in weekly median Rt at the match date between the exposed county and the matched unexposed county must be below 0.30, a threshold informed by research conducted by Huang et al.[b]
**School sports activity**	School sports activity data were obtained from a systematic scan of official school district website content, including athletics calendars, COVID-19 reopening plans, district announcements, or linked official Twitter and Facebook announcements. Schools were considered to have active fall sports activities if school sanctioned sports teams were holding practices or competing in games between August 1 and October 31, 2021.	The matched unexposed county must have the same plan for school-district-level fall sports activities as the exposed county.

[a] Cori A, Ferguson NM, Fraser C, Cauchemez S. A new framework and software to estimate time-varying reproduction numbers during epidemics. Am J Epidemiol. 2013;178(9):1505-1512. doi:10.1093/aje/kwt133

[b] Huang J, Fisher BT, Tam V, et al. The Effectiveness Of Government Masking Mandates On COVID-19 County-Level Case Incidence Across The United States, 2020. Health Aff (Millwood). 2022;41(3):445-453. doi:10.1377/HLTHAFF.2021.01072/ASSET/IMAGES/LARGE/FIGUREEX3.JPEG

© 2023 Matone M et al. *JAMA Network Open*.

eTable 2. Covariates Adjusted in Regression Models

Covariates		Data source and processing
Time-invariant:		
	Proportion of county residents below 200% of poverty level	American Community Survey
	Proportion of county residents with diabetes	Behavioral Risk Factor Surveillance System
	Mask wearing in the general population	Data on mask use comes from interviews conducted online at the request of The New York Times during that time period. Specifically, each participant was asked "How often do you wear a mask in public when you expect to be within six feet of another person" between July 2 and July 14 of 2020.[a] Percent of respondents self-reporting to 'never' wear a mask in the public in each county was adjusted as a covariate.
Time-varying (Daily values on the following covariates were adjusted):		
	Temperature	Wet-bulb temperatures, obtained from the National Oceanic and Atmospheric Administration Local Climatological Data, were used to capture the complex thermodynamic relationship between temperature and humidity. Rolling averages of wet-bulb temperatures within 4-14 days before COVID-19 case identification were used to account for the incubation period of SARS-CoV-2 prior to case identification, informed by Rubin et al.[b] Piecewise linear terms were used to fit the nonlinear effect of wet-bulb temperatures on daily new cases county of COVID-19, with a breakpoint of 11°C informed by Jing et al.[c]
	Social distancing or mobility measures	These measures were adjusted in Sensitivity Analyses. See eMethod, eResults, eTable3, and eTable4 for details.

[a] Detailed Map of Who Is Wearing Masks in the U.S. - The New York Times. Accessed May 26, 2021. https://www.nytimes.com/interactive/2020/07/17/upshot/coronavirus-face-mask-map.html
[b] Rubin D, Huang J, Fisher BT, et al. Association of Social Distancing, Population Density, and Temperature with the Instantaneous Reproduction Number of SARS-CoV-2 in Counties across the United States. JAMA Netw Open. 2020;3(7):2016099. doi:10.1001/jamanetworkopen.2020.1609
[c] Huang J, Fisher BT, Tam V, et al. The Effectiveness Of Government Masking Mandates On COVID-19 County-Level Case Incidence Across The United States, 2020. Health Aff (Millwood). 2022;41(3):445-453. doi:10.1377/HLTHAFF.2021.01072/ASSET/IMAGES/LARGE/FIGUREEX3.JPEG

© 2023 Matone M et al. *JAMA Network Open*.

eMethods. Sensitivity Analyses

We conducted multiple sensitivity analyses to evaluate the robustness of our effect estimates. In **sensitivity analysis #1,** we used a stricter constraint on the school reopening date of the unexposed counties, by only including matched pairs in which the unexposed county did not reopen schools within 5 weeks (primary analysis used 4 weeks) after the exposed county's reopening date. In this way, we had less overlapping of school-reopened days between matched exposed and unexposed counties in the observation period. In **sensitivity analysis #2**, we only included matched pairs in which the exposed county reopened schools in the August of 2020. In this analysis, the calendar time of the observation periods (2 weeks prior - 8 weeks post school reopening date) was similar for all pairs of counties and was before the holiday season of 2020. The **sensitivity analysis #3** only included matched pairs in which the inter-county geographic distance is >50km and <500km. Matched pairs that are too close to each other (<=50 km) were excluded to account for the possible between-county correlation in community COVID-19 transmission, and matched pairs that are too far away from each other (>500km) to account for residual confounding in temperature and unmeasured confounding due to heterogeneity between counties. In sensitivity analysis #4, we considered the potential bias of over-representing case incidence from matched control counties due to our sampling with replacement matching algorithm. Because we used matching with replacement in selecting the matched controls, one unexposed county could be matched with multiple exposed counties. In this analysis, to avoid our results being heavily impacted by a few unexposed counties that were over-represented among the controls, we only included matched pairs in which unexposed counties were only permitted to match to 3 or fewer exposed counties.

Finally, in **sensitivity analysis #5**, we examined whether the effect of school reopening was confounded by the change in social distancing in communities over time. A series of models were fitted with and without adjustment for the variables that encoded daily county-level measures of social distancing, including community and workplace mobility, over time. Several social distancing and mobility measures from two data sources were considered as potential confounders: 1) percent change in visitation to non-essential venues compared to a pre- COVID-19 period; 2) visitation to retail and recreation places; 3) visitation to parks; and 4) mobility to workplaces. The first, percent change in visitation to non-essential venues was measured using daily cellphone movement provided by Unacast (COVID-19 Social Distancing Scoreboard — Unacast. Accessed May 26, 2021. https://www.unacast.com/covid19/social-distancing-scoreboard). Essential venues (e.g., food stores, pharmacies) and non-essential venues (e.g. cinemas and theaters, spas and hair salons, hotels, restaurants) were categorized based on guidelines issued by various state governments and policy makers (A Guide to State Coronavirus Reopenings and Lockdowns - WSJ. Accessed May 26, 2021. https://www.wsj.com/articles/a-state-by-state-guide-to-coronavirus-lockdowns-11584749351). The change in visitation to non-essential venues was calculated by comparing the daily visitation during observation period compared to the average visitations in a four-week pre-pandemic baseline period (February 10th and March 8th, 2020) in each county. The additional measures of social distancing related to visitation to community and workplace settings and were obtained from Google COVID-19 Community Mobility Report. These measures characterize how visits and length of stay at different places change based on data from users who have opted-in to Location History for their Google Account, so the data represents a sample of Google users in each county. The measures were defined as percent change in daily visits compared to pre-COVID baseline, which was the median value, for the corresponding day of the week, during the 5-week period of January 3rd to February 6th, 2020.

© 2023 Matone M et al. *JAMA Network Open*.

eTable 3. Sensitivity Analyses: Ratios of Daily COVID-19 Incidence per 100 000 Individuals Between Matched Counties With or Without School Reopening, in Subgroups of Matched Pairs

Subgroups of matched pairs		**Adjusted daily case counts ratio (95% CI) between exposed and unexposed counties after school reopening [a]**				
		On 14 days post	On 28 days post	On 42 days post	On 56 days post	P-value [d]
1	**Among matched pairs in which the unexposed county did not reopen schools within 5 weeks after the exposed county's reopening date** (41 pairs, 67 counties)	1.11 (0.89, 1.37)	1.23 (0.96, 1.58)	1.31 (1.02, 1.71)	1.30 (1.01, 1.69)	0.017
2	**Among matched pairs in which the exposed county reopened schools in the August of 2020** (38 pairs, 60 counties)	1.16 (0.93, 1.45)	1.28 (0.98, 1.66)	1.41 (1.07, 1.86)	1.55 (1.18, 2.04)	0.007
3	**Among matched pairs in which the inter-county geographic distance is >50km and <500km [b]** (42 pairs, 66 counties)	1.16 (0.95, 1.41)	1.23 (0.97, 1.56)	1.28 (0.999, 1.64)	1.29 (1.01, 1.65)	0.051
4	**Among matched pairs in which unexposed counties were matched to <=3 exposed counties [c]** (46 pairs, 75 counties)	1.21 (0.996, 1.47)	1.25 (0.996, 1.57)	1.29 (1.02, 1.64)	1.37 (1.08, 1.74)	0.018
5	**Among matched pairs within the Southeast Region** (45 pairs, 69 counties)	1.11 (0.91, 1.35)	1.24 (0.99, 1.56)	1.34 (1.06, 1.70)	1.33 (1.05, 1.67)	0.0021

[a] The effect estimates were from generalized linear mixed effect models (GLMM). The models adjusted for population density, percent of population under age 18, rate of poverty, use of masks in public (percent of people reporting to always wear mask in public when expecting to be within six feet of another person, in a survey during July 2-14 of 2020), and daily wet-bulb-temperature (3-14 days lagged rolling averages). The GLMM used a log link function to account for the skewed distribution of daily cases in each county. Hierarchical random intercept and slope were used to account for correlations within matched pairs of counties and the correlations within longitudinal measures of individual county, separately.
[b] The inter-county geographic straight-line distance was calculated using Esri ArcGIS Pro (Redlands, CA).
[c] Because we used matching with replacement in selecting the matched controls, one unexposed county can be matched with multiple exposed counties.
[d] P values obtained from testing for null hypothesis of no significant difference in trajectory of incidence case over time between exposed and unexposed counties, using Wald test on the regression coefficients of
three interactions between school reopening and polynomials of time (linear, quadratic, and cubic time).

© 2023 Matone M et al. *JAMA Network Open.*

eTable 4. Ratios of Daily COVID-19 Incidence per 100 000 Individuals Between Matched Counties With or Without School Reopening, in Models Further Adjusting for Social Distancing and Mobility Measures

Model	Predictors and Covariates	Adjusted daily case counts ratio (95% CI) between exposed and unexposed counties after school reopening [a] (in 51 matched pairs of counties)			
		On 14 days post	On 28 days post	On 42 days post	On 56 days post
**Model 0 (Un-adjusted)**	Time + School reopening + time*school reopening	1.10 (0.91, 1.31)	1.19 (0.96, 1.47)	1.29 (1.03, 1.60)	1.36 (1.10, 1.69)
**Model 1**	Model 0 + Population Density + Population under 18 + Rate of Poverty + Mask use + Temperature [b]	1.06 (0.89, 1.28)	1.15 (0.93, 1.42)	1.24 (1.001, 1.55)	1.31 (1.06-1.62)
**Model 2**	Model 1 + visitation to non-essential venues [c]	1.04 (0.87, 1.26)	1.13 (0.91, 1.41)	1.23 (0.98, 1.54)	1.31 (1.04, 1.64)
**Model 3**	Model 1 + visitation to retail & recreation places [d]	1.06 (0.88, 1.27)	1.16 (0.94, 1.43)	1.28 (1.03, 1.59)	1.37 (1.11, 1.70)
**Model 4**	Model 1 + visitation to parks) [d]	0.99 (0.80, 1.23)	1.08 (0.84, 1.40)	1.22 (0.93, 1.59)	1.38 (1.06, 1.80)
**Model 5**	Model 1 + mobility to workplaces) [d]	1.02 (0.85, 1.22)	1.10 (0.89, 1.35)	1.20 (0.96, 1.49)	1.29 (1.04, 1.58)

[a] The effect estimates were from generalized linear mixed effect models (GLMM). The GLMM used a log link function to account for the skewed distribution of daily cases in each county. Hierarchical random intercept and slope were used in all models (Models 0-5) to account for correlations within matched pairs of counties and the correlations within longitudinal measures of individual county, separately.
[b] Model 1 adjusted for population density, percent of population under age 18, rate of poverty, use of masks in public (percent of people reporting to always wear mask in public when expecting to be within six feet of another person, in a survey during July 2-14 of 2020), and daily wet-bulb-temperature (3-14 days lagged rolling averages).
[c] Model 2 further adjusted for a social distancing measure, defined as the percent change in visitation to non-essential venues compared to a pre- COVID-19 period. Essential venues (e.g. food stores, pharmacies) and non-essential venues (e.g. cinemas and theaters, spas and hair salons, hotels, restaurants) were categories based on guidelines issued by various state governments and policy makers, and visitation to non-essential venues was measured using daily cellphone movement provided by Unacast. The change in visitation to non-essential venues was calculated by comparing the daily visitation during observation period compared to the average visitations in a four-week pre-pandemic baseline period (February 10th and March 8th, 2020) in each county. 3-14 days lagged rolling averages of this measure was used in the model.
[d] In Models 3-5, visitation to retail & recreation places, visitation to parks, and mobility to workplaces were obtained from Google COVID-19 Community Mobility Report. These measures show how visits and length of stay at different places change based on data from users who have opted-in to Location History for their Google Account, so the data represents a sample of Google users in each county. The measures were defined as percent change in daily visits compared to pre-COVID baseline, which was the median value, for the corresponding day of the week, during the 5-week period Jan 3–Feb 6, 2020.

© 2023 Matone M et al. *JAMA Network Open*.
