# Supplementary material for: Association of In-person vs Virtual Education With Community COVID-19 Case Incidence Following School Reopenings in the First Year of the COVID-19 Pandemic
Source: JAMA Netw Open. 2023 Apr 14;6(4):e238300. doi: 10.1001/jamanetworkopen.2023.8300 (PMC10105309; doi:10.1001/jamanetworkopen.2023.8300)
Supplement: Supplement 2. — Data Sharing Statement [file jamanetwopen-e238300-s002.pdf]

Data Sharing Statement

Matone. Association of In-person vs Virtual Education With Community COVID-19 Case Incidence Following School Reopenings in the First Year of the COVID-19 Pandemic. *JAMA Netw Open*. Published April 14, 2023. doi:10.1001/jamanetworkopen.2023.8300

Data
Data available: Yes
Data types: Data (not involving human participants), Data dictionary
How to access data: matonem@chop.edu
When available: beginning date: 03-01-2023

Supporting Documents
Document types: None

Additional Information
Who can access the data: Anyone
Types of analyses: For any purpose
Mechanisms of data availability: Data access agreement
